# Spatial confidentiality and GIS: re-engineering mortality locations from published maps about Hurricane Katrina

**DOI:** 10.1186/1476-072X-5-44

**Published:** 2006-10-10

**Authors:** Andrew J Curtis, Jacqueline W Mills, Michael Leitner

**Affiliations:** 1 World Health Organization Collaborating Center for Remote Sensing and GIS for Public Health, Department of Geography and Anthropology, Louisiana State University, Baton Rouge, USA; 2 LSU GIS Clearinghouse Cooperative, Disaster Science Management Louisiana State University, Baton Rouge, USA

## Abstract

**Background:**

Geographic Information Systems (GIS) can provide valuable insight into patterns of human activity. Online spatial display applications, such as Google Earth, can democratise this information by disseminating it to the general public. Although this is a generally positive advance for society, there is a legitimate concern involving the disclosure of confidential information through spatial display. Although guidelines exist for aggregated data, little has been written concerning the display of point level information. The concern is that a map containing points representing cases of cancer or an infectious disease, could be re-engineered back to identify an actual residence. This risk is investigated using point mortality locations from Hurricane Katrina re-engineered from a map published in the Baton Rouge Advocate newspaper, and a field team validating these residences using search and rescue building markings.

**Results:**

We show that the residence of an individual, visualized as a generalized point covering approximately one and half city blocks on a map, can be re-engineered back to identify the actual house location, or at least a close neighbour, even if the map contains little spatial reference information. The degree of re-engineering success is also shown to depend on the urban characteristic of the neighborhood.

**Conclusion:**

The results in this paper suggest a need to re-evaluate current guidelines for the display of point (address level) data. Examples of other point maps displaying health data extracted from the academic literature are presented where a similar re-engineering approach might cause concern with respect to violating confidentiality. More research is also needed into the role urban structure plays in the accuracy of re-engineering. We suggest that health and spatial scientists should be proactive and suggest a series of point level spatial confidentiality guidelines before governmental decisions are made which may be reactionary toward the threat of revealing confidential information, thereby imposing draconian limits on research using a GIS.

## Background

Geospatial technologies and even Internet applications such as Google Earth are now frequently used in both social and biological sciences in the search for spatial patterns and processes (for recent commentaries and examples see [1-3]). Geospatial display on the internet, such as Google Earth, not only provides a means to publicize the importance of "geography", but also acts as a dissemination tool for spatial results. This democratisation of spatial insight can have a dramatic impact on communities without the technical ability, hardware or software to use a Geographic Information System (GIS). At a recent symposium jointly hosted by the National Institute on Drug Abuse and the Association of American Geographers [4], in the concluding discussion session the universal appreciation of GIS was obvious. However, there was also a general concern expressed about preserving individual confidentiality within spatial displays. This concern is justified as map making, and the ability to deliver maps to a mass audience through the Internet becomes steadily easier [5-8].

Most health related maps are thematic involving data aggregated to a spatial unit, the most common map type being the graduated color or "choropleth" map. In an effort to protect individuals, the Health Insurance Portability and Accountability Act (HIPAA) provides guidelines to inform researchers as how to preserve confidentiality by employing minimum spatial denominator units on a map. According to the U.S. Department of Health and Human Services (HHS) health information can only be disclosed, if all zip codes with the same three initial digits exceed 20,000 people; otherwise the initial three digits are changed to 000 [9]. This guideline can be interpreted in another way. If we (conservatively) assume each building contains approximately 4 people, then health information should not be mapped at the residential level in areas containing less than five thousand buildings. It is unfortunate, however, that little exists explicitly for data display at this point (residential) level, even though many such maps exist in the academic literature. Examples of these health related point level maps which will be briefly discussed in this paper include, the spatial association between birth outcomes and disease (Toxoplasmosis) [10], birth outcomes and residential/work proximity to the World Trade Center [11], health effects of living close to heavily trafficked routes [12] and cases of an infectious disease[13].

Among the precautions that can be taken to preserve point level confidentiality include the masking or spatial manipulation of the location [14-17], the removal of other geographic reference layers, or the use of software agents [18]. In this last example the investigator never works with point level information. A software agent acting on behalf of the investigator, can access the data server where confidential data are stored, perform the required analysis functions, and return only useful aggregate results without any individual-identifiable details to the researcher [18].

If the researcher does have access to the original data, simply removing map detail, for example a road network, may not be enough to ensure that confidentiality is preserved [16]. For example, if a residential "point" appears in the middle of a map displaying only zip code boundaries, how could this display violate any individual's confidentiality, especially if all zip codes contain at least 20,000 individuals? The problem arises if the zip code boundaries can be used to re-engineer the map data back to smaller neighbourhoods, maybe even a street or single house, and in so doing dramatically change the size of the denominator. It is only prudent to test this assumption, and attempt to re-engineer information (also called inverse or reverse address matching) back to an individual residence from an apparently "detail free" map [16]. This is especially important given how easy it is to output GIS layers and display information to a large Internet audience through geospatial packages such as Google Earth, layers which as graphics can in turn be extracted and imported back into a GIS environment.

Contributing factors in the successful re-engineering of information from a cartographic display is the published map's scale, the size (and quality) of the published map, the projection used, and the accuracy (or error) in the initial mapping of the points. An error one would expect to find between the geocoded and re-engineered address is the positional error due to the address-matching procedure. This error occurs when a list of addresses is matched to a street network layer using a GIS. The extent of this error can be calculated by comparing the location of the gecoded addresses with a second measurement, usually generated with a Global Positioning System (GPS) satellite receiver or from an aerial image. As an example of such an investigation using a random sample of 200 addresses taken from a life history project of 3286 subjects, Bonner et al. (2003) found 79% of all distances between the gecoded and the GPS point to be within 100 m, the median distance being 38 m. [19]. The same study also found that urban addresses were slightly more accurate than non-urban, with 33% of addresses being within 25 meters. The accuracy of placement also varied according to the length of the road, with longer road segments, which again tend to be found in non-urban areas, being the least accurate [19].

When using a GIS to investigate geocoding error, the accuracy of the GPS measurement should also be taken into consideration. The positional accuracy of the GPS receiver can be tested using a National Geodetic Survey (NGS) point. GPS positions are recorded by holding the unit directly over the NGS point, for which the exact location is known. Usually, more than 100 positions are recorded for the same location at equal time intervals (for example, every second). The final coordinate is then calculated as the spatial average of all recorded positions. Positional data can be used uncorrected or differentially corrected with data from a nearby base station. Differentially corrected positions have a higher accuracy compared to uncorrected positions. Listi, et al. (2007) tested the positional accuracy of the GeoExplorer^® ^3 Data Collection System (a hand-held GPS receiver in the mid-price range) from Trimble Navigation Limited for field mapping scattered human remains or other materials in forensic investigations. Using the spatial average of 206 positions and without any differential correction, the GPS unit produced an error of 3.523 meters (approx. 11.62 feet). In contrast, post-processed differential correction for the same spatial average of 206 positions produced an error of 0.424 meters (approx. 1.4 feet))[20]. Other considerations when using a GPS to confirm geocode accuracy include where the measurement was taken (for example the property line or front door), the position of the satellites, atmospheric conditions, and the line-of-sight to the satellites, which can be interrupted by tree cover, buildings or other structures.

As an alternative measure of geocode accuracy, Cayo and Talbot (2003) determined the positional error for 3,000 residential addresses using the distance between each geocoded point and its true location as determined with aerial imagery [21]. They found error increased as population density decreased and that the geocoding error substantially decreased, when property data are used instead of street network files. Both GPS and aerial imagery will be used in this paper to verify re-engineered addresses.

**Figure 1:**
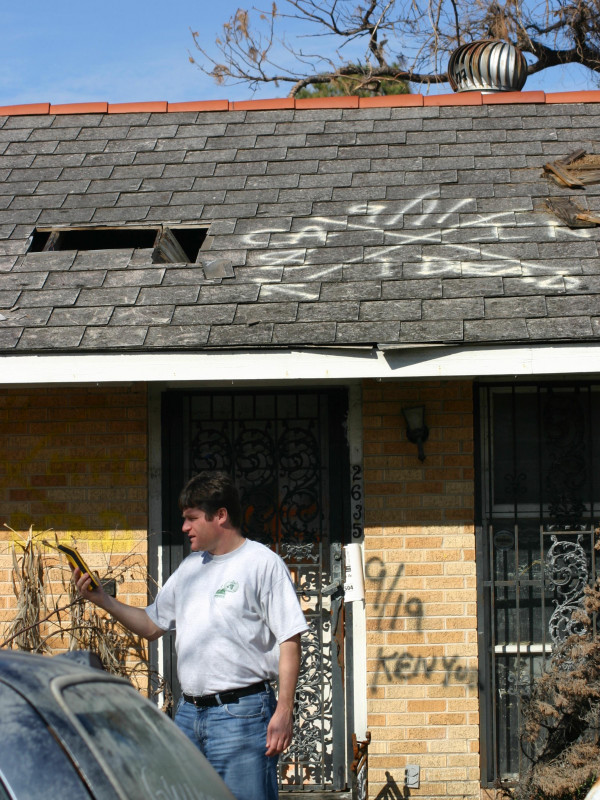
**Hurricane Katrina search and rescue marking**. This house displays the typical search and rescue "X". A California task force visited the house on September 11^th ^and they found "1 dead". "Kenyon" removed the body on September 19^th^. The field team member is seen in front of the house marking its location with a GPS.

The question posed in this paper is to what degree can confidential information in the form of a person's home residence be extracted, or "re-engineered" from a map appearing in a journal article, book, newspaper or Internet site, especially if most traditional spatial reference layers, such as road networks, are removed from the map? In order to investigate this question two experiments could have been designed. The first would have been to fabricate data that are then geocoded, mapped, and printed before being given to a second team as a hard copy for re-engineering. First (fabricated) and second (re-engineered) addresses could be compared for separating distance (see [22]for an example). This is a valuable line of inquiry because of the insights it might provide not only in terms of the violation of confidentiality, but also in how the underlying population structure, street type, and building patterns impact the process of re-engineering. In his experiment Armstrong (2002) found that 68% of addresses could be re-engineered to the correct residence, 85% to the immediate neighbour, and 97% to the correct street segment [16]. He goes on to comment that errors in the geocode process would always leave an element of doubt in this type of exercise as the accuracy (was it the "correct" house) would be fuzzy. The second approach, and the one employed in this paper, is to take an actual published map of confidential residential information, and re-engineer these data back to actual addresses. This approach is the most appealing as it approximates the danger posed by spatially representing confidential information, and having a third party with no access to the actual data attempting to identify the residence of the "case". Although the second approach is most appealing, it is, by definition, almost impossible to investigate. If the data being mapped are confidential, how would the field researcher know if he/she had discovered the actual residence? However, Hurricane Katrina provided such an opportunity as a published map displayed point locations of deaths, and the search and rescue notations spray-painted on the houses allowed field teams to verify the accuracy of the re-engineering process. These markings also provide the means to identify the correct address missing in the Armstrong study. Figure 1 shows a field team member standing in front of a typical destroyed house using a GPS to mark the location. The search and rescue markings can be seen on both the roof and wall of the building. These markings can be deciphered as follows: the search team affiliation (left side of the X), the date of visit (top of the X), any "additional" information such as signs of looting or whether animals were present (right side of the X), and if any corpses were discovered (bottom of the X). It was therefore possible to identify those houses where fatalities had been discovered if a "1" or greater number was marked at the bottom of the "X". In addition, other markings are associated with mortalities, including comments such as "1 DB in back" or "Ken" which identifies the recovery team most frequently tasked to remove the body. In Figure 1 the first search and rescue team identified a dead body on September 11^th ^("1 dead" appears at the bottom of the X), and the body was removed on September 19^th^.

**Figure 2:**
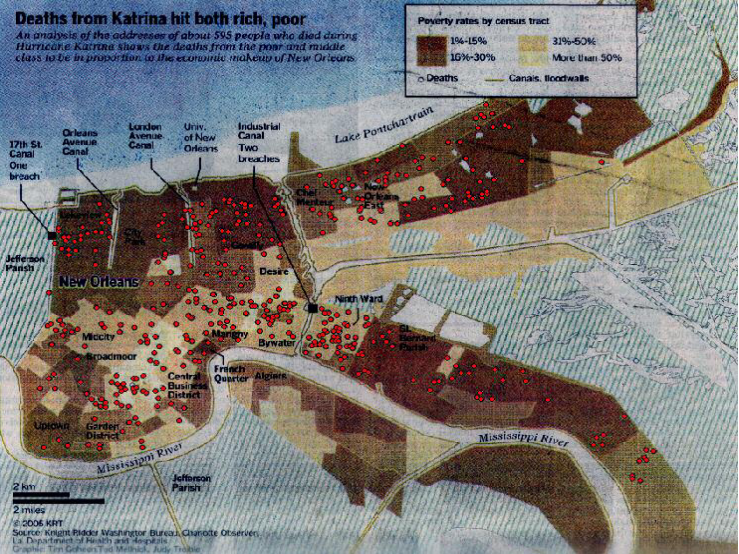
**Deaths from Katrina map**. The original map appearing in the Baton Rouge *Advocate *on December 30^th^. The red points are the mortality locations which have been digitised and overlaid on the original image.

The Baton Rouge *Advocate *printed a map of mortality locations in Orleans and St Bernard Parishes on the 30^th ^December 2005 (Fig 2). This map contained neighborhood areas of New Orleans (for example the Garden District, Lower Ninth Ward) and important features of the disaster, including the location of levee breaks, canals and floodwalls. The mortality locations were overlaid on a graduated color surface of poverty rate mapped by census tract. The map contained no roads or other references points, except for a generalized location of the University of New Orleans. Three neighborhoods heavily impacted by Hurricane Katrina, but of different urban character, were chosen for this study, these being the area around the London Canal levee break, New Orleans East, which suffered flooding directly from Lake Pontchartrain's surge, and the Lower Ninth Ward which flooded as a result of the Industrial Canal break. The specific goal of this investigation was to use the *Advocate *map to guide field teams to the actual residence where a body was found. The larger concept was to investigate how a similar map displaying health outcomes could also be re-engineered to an actual residence. It can be argued that the *Advocate *map of mortalities does not represent a confidential surface as these data were mapped in the local paper, they are still "officially" considered such and have not been released to the authors of this paper for further validation purposes. However, this argument does not detract from the larger purpose of the study.

## Results

Table One displays the distances from all mortalities re-engineered from the *Advocate *map to the closest street segment of the Orleans Parish street network. This was performed to assess the degree to which the underlying street pattern was preserved in the point locations on the map, remembering that actual streets had been removed. In total over 22% of all re-engineered mortalities were within 5 meters of a street segment. This percentage rose to over 45% when the distance from the mortality coordinate to the street was 10 meters or less. This result suggests that the original cartographer had employed a GIS based address matching approach and the underlying street pattern was still preserved within the mortality distribution. Of the three study neighborhoods investigated in this paper, the London Canal area had the greatest percentage of addresses within 5 meters (37.5%) of a road section. Although this might be indicative of the urban character of the neighborhood, with more tightly packed streets leading to a shorter distance to a road section by chance alone, the percentage of randomly generated points from 100 simulation runs in the same area within 5 meters was only 18%.

For all of Orleans Parish 18.4% of re-engineered mortalities were greater than 25 meters from a street centre line. Of the three study neighborhoods, the area with the highest percentage of (poorly) re-engineered mortalities falling in this 25-meter category was New Orleans East (25%), with the smallest percentage being the London Canal area (4.2%). When considering the randomly generated points falling into this greater than 25-meter category, all three neighborhoods registered higher percentages, ranging from 42% in New Orleans East to 25.9% in the Lower Ninth Ward. This shows how the underlying street pattern was still preserved in the mapped mortality surface.

**Figure 3:**
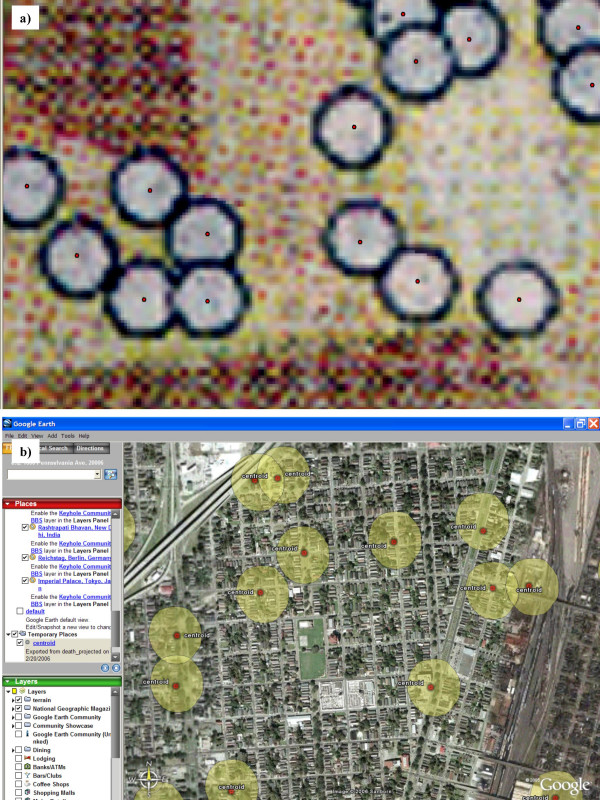
**Digitised mortality locations**. Mortality locations have been digitised from the newspaper map and are shown in terms of the coarseness of the original map image (a), and on a Google Earth display using the kmler tool in ArcMap (b). Each circle covers approximately 1.5 city blocks.

Of the 24 mortalities re-engineered from the *Advocate *map in New Orleans East, 16 were identified to actual houses by the field team. Around these 16 houses a further eight residences were also sprayed with mortality markings. These additional residences were so close to the re-engineered location so as to fall within the "mortality circle" which covered approximately one-and-half-city blocks (Fig 3). The "mortality circle" is the white dot displaying the death location on the *Advocate *map. Of the 20 mortalities re-engineered from the *Advocate *map in the London Canal area, 14 were identified by the field team. Around these 14 houses a further two residences also could be identified with mortality markings. Of the 36 mortalities re-engineered from the *Advocate *map in the Lower Ninth Ward, 22 were identified by the field team. Around these 22 houses a further four residences could also be identified with mortality markings.

Table Two displays distances between the re-engineered mortalities and the closest field verified residences. The percentage of verified residences falling into each category of 5-meter increments is displayed. Of the three neighborhoods, New Orleans East produced the highest percentage of close distances between the re-engineered mortality and the actual residence "pairs" with almost 23% being within 10 meters, and over 40% being within 20 meters. By comparison, only 6% of the pairs for the London Canal, and 23% for the Lower Ninth Ward were within 20 meters.

**Figure 4:**
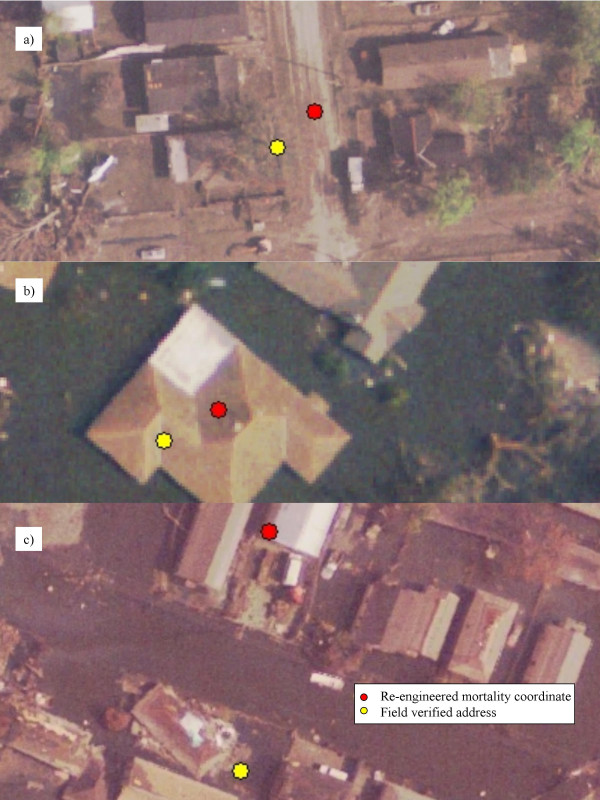
**Distances between re-engineered mortalities and verified locations**. The two locations in the detail from New Orleans East are separated by half a street width (a). In the London Canal Area, both locations fall on the same house (b). In the Lower Ninth Ward the two locations are found on either side of the same street (c).

As previously mentioned in this paper, there is an element of uncertainty concerning any exact distance measurement due to variations in both geocoding and GPS data collection. Therefore a second measure, the number of actual houses separating the re-engineered mortality and the field verified residence, as identified on the aerial imagery, was recorded. In three instances for New Orleans East both the re-engineered mortality and the field verified residence fell on the same house. In addition, three further pairs were separated only by the distance of the house to the middle of the road. Five locations were next door, and three more had only two intervening residences. The greatest intervening distance was six residences. For the London Canal area, one of the re-engineered mortalities fell on the same field-verified residence, three were separated only by the distance to the middle of the road or the house on the opposite side of the street, and two houses were immediate neighbours to the re-engineered location. The greatest separating distance was nine houses. The extent of damage in the Lower Ninth Ward made this form of measurement impossible due to the amount of residential destruction with many houses having floated from their original foundation. Figures 4a–c display details for all three neighborhoods, with the red dot marking the re-engineered coordinate, and the yellow dot being the GPS measurement. Figure 4a (New Orleans East) shows an example of the separating measure between the pairs as being the middle of the road, figure 4b (London Canal) is an example of where both coordinates fall on the same residence, and 4c (Lower Ninth Ward) shows where the address is on the other side of the street.

A final step was to determine if the re-engineered coordinate actually guided the field team to the mortality location, or whether chance alone would have resulted in the same level of discovery. A series of random coordinates equalling the number of mortality residences were scattered throughout the study areas. This simulation was repeated 100 times generating a test distribution of mortalities. A 95% confidence level was determined if the distance between the actual re-engineered mortality and the field verified residence was smaller than in 95 of all simulated distances between a random coordinate and the same field verified residence. Meeting this 95% level were 73% of the New Orleans East pairs, 75% of the London Canal pairs, and 50% of the Lower Ninth Ward pairs. As an even more extreme comparison, for New Orleans East 9 of the 22 pairs were closer than in any of the simulation runs. Similarly, 3 of 16 pairs for the London Canal area, and 1 in the Lower Ninth Ward were closer than to any of the simulated coordinates.

## Discussion

The success of this research should not be judged by the percentage of successfully re-engineered mortalities that can be verified back to an actual residence as other externalities could impact this process. These include: the body was recovered from a non-residence, such as a road median; the house has since been cleaned of all markings, or no distinguishable "mortality" marking was left on the residence; and the neighborhood could have suffered such extreme damage that mortality markings were not obvious, or the residence itself had disappeared or been moved. The success of re-engineering mortalities from the *Advocate *map should rather be judged if *any *residence could be verified. The fact that many of the re-engineered coordinates could be used to identify an actual address, or an address within the immediate vicinity, should sound a note of caution for academics publishing maps displaying human cases as points. In order to further impress on this point, and to show that similar cartographies have been employed to map health data, the following surfaces in the *American Journal of Public Health, American Journal of Tropical Medicine and Hygiene, Emerging Infectious Diseases, Environmental Health Perspectives*, and the *International Journal of Health Geographics *are briefly discussed.

Oyana et al. (2006) consider the proximity of asthma cases and controls to different pollution sources [12]. Their map contains an outer boundary shape of Buffalo, New York. The map contains additional "reference" material in terms of major roads. Cases (and controls) are displayed as a triangle or dot. The inner area of the study, the "West Side" is heavily populated making the potential re-engineering of addresses difficult. However, the re-engineering process would be easier to accomplish in the more sparsely populated areas (in terms of cases and controls), and especially if the residence falls close to locations useful for registering the image. For example, the area to the south of the map would be of particular concern with relatively few cases, and where several roads converge.

Lederman et al (2004), in their investigation of the effects of the World Trade Center disaster on birth outcomes, create a map showing work and home addresses as points [11]. Geographic features in the map that would allow for the georegistering of the image include the land/water boundary and major roads. Although the map on the journal page is relatively small, it is also possible to view a larger version of the figure at the journal's website.

Eng et al (1999) use three maps in their study of toxoplasmosis on Vancouver island, British Columbia, with residences being displayed as points [10]. The first map displays the geographic location of 94 acute cases of toxoplasmosis. The second and third maps display the location of women screened during pregnancy, who were either negative, or had non acute toxoplasmosis. These maps appear to be relatively safe as they contain few geographic references suitable for the georegistering of the image, beyond a detailed outer boundary of the map. As a larger version of the map is available on the journals website, it would still be interesting to see how close a re-engineered coordinate would be to the actual address.

Huhn et al (2005), in their investigation of the 2002 West Nile virus epidemic of Illinois, use a map displaying West Nile virus cases in Cook County. A solid cross is used to mark the addresses of 536 cases. Although the overlap of crosses in the more case clustered areas would make the re-engineering of individual residences difficult, there are several sections of the map where relative geographic isolation of cases occurs [13]. In a second publication investigating the same outbreak, cases are overlaid onto a raster image of elevation [23]. This raises another issue, how would the re-engineering process be improved if a commonly available grid of data is used as backdrop? A second map in the same article is the most obvious candidate for a successful re-engineering as human cases are displayed as crosses on a map of census tracts. This map contains a greater georegistration potential than the *Advocate *New Orleans map used in this paper.

In none of these examples is mention made of any masking procedure applied to the point placement of human cases on the map. This suggests that the cases (usually shown as points) should mirror the underlying street network, and are therefore vulnerable to re-engineering. These comments are not meant to be criticisms of the academics involved in each study as the danger of re-engineering information from a map is a relatively new concern, though warnings have previously been sounded about mapping unmodified geocoded data [16,24]. There are, however, other more proactive studies that should be applauded for addressing confidentiality in their display.

Rothenberg et al. (2005) map the social and geographic interconnections for a subgroup of HIV infected individuals in their Colorado Springs study using a spider plot (nodes being connected with lines, with color being added to indicate the strength of connection). The authors comment that "...the map has insufficient detail to read the exact placement of nodes." [26] However, in order to preserve confidentiality each node was randomly moved by 1600 m. The authors further state that this "masking" allows for easier map interpretation while preserving the geographic relationship between nodes and links. Although the authors were mistaken in that the map does contain sufficient detail to allow re-engineering (census block boundaries are included), the random displacement of the nodes makes this a mute point.

Previous research has shown that urban density, urban/non-urban, and even length of street segment impact the success of geocoding [19], and similarly this paper has revealed how urban neighborhood structure plays a marked role in the success of re-engineering residential information. This is largely a result of two factors: the housing pattern on a street, which can affect address-matching results, and the amount of neighborhood detail allowing for more accurate georegistering of the image.

For example, the London Canal area has the highest percentage of mortalities (37.5) being close (within 5 meters) to a street section (Table 1). This area also has the lowest percentage (4.2%) of locations falling in the greatest distance away from a street category (above 25 m). If we look at the original georeferenced map, the London Canal area has the greater number of adjoining Census tracts of the three neighborhoods, resulting in more geographic detail, which in turn allows for more accurate georegistering of that part of the image. Although these coordinates are close to the street network, this does not reflect the accurate placement of geocoded points along a street, which in turn would have an impact in terms of the distance between mortality coordinate and verified residence. New Orleans East was the most successful in terms of this measure with just over 22% of the pairs within 10 meters. This suggests that house spacing within this area allows for a more accurate geocode. Most address matches are calculated with the house number being proportionally placed within the range of addresses on a street segment. More accurate address placement tends to occur in homogenous neighborhoods, such as in the suburbs, or in newer housing developments such as in New Orleans East. The worst neighborhood for geocode accuracy is the Lower Ninth Ward, with no pairs being within 10 meters. This can be explained by the considerable heterogeneity of housing structure, both in terms of size and lot placement. However, even in this neighborhood, a high proportion of the re-engineered mortalities were found, and 50% of these were closer than to any simulated "address" in 95 out of 100 simulation runs.

**Table 1:** Distance for re-engineered and randomly generated points to the closest road.

Distance from road (meters)	0 to 5	6 to 10	11 to 15	16 to 20	21 to 25	Above 25
New Orleans East						
Deaths From Map	16.7%	16.7%	8.3%	25.0%	8.3%	25.0%
Random Points	16.4%	12.5%	10.8%	9.4%	9.0%	42.0%
						
London Canal						
Deaths From Map	37.5%	4.2%	16.7%	25.0%	8.3%	4.2%
Random Points	18.0%	15.7%	13.0%	10.3%	11.0%	32.1%
						
Ninth Ward						
Deaths From Map	19.4%	27.8%	16.7%	11.1%	8.3%	16.7%
Random Points	19.6%	18.0%	14.9%	11.8%	10.0%	25.9%
						
All Deaths From Map (Orleans Parish)	22.5%	23.0%	14.1%	13.6%	8.4%	18.4%

## Conclusion

This paper has shown that any map containing point data, even when little secondary spatial information is presented, is vulnerable to being re-engineered to reveal the actual addresses associated with the points. It is therefore vital that some masking occurs of the original point data. Although HIPAA regulations state that health information can only be disclosed, if all zip codes with the same three initial digits exceed 20,000 people it is still feasible that a point displayed on a Parish boundary with no political subdivisions, meaning the cartographer is not violating any HIPAA regulation in terms of an apparent minimum denominator, could still be re-engineered if enough detail is present in the boundary shape. The question needing further discussion is how we should determine minimum denominators. If such a re-engineering process places a residence within a denominator area of 50 houses, this is a violation of the spirit of HIPAA.

Further research should concentrate on the degree of masking required in relation to urban structure, what could be considered safe amounts of map detail, and an appropriate minimum denominator of "alternative" residences. The suggestion should also be that until such research has been conducted, are maps really necessary in publications? Why not chose an abstract space on which to display spatial patterns [25]. The reader of the paper may need a graphic to understand the described relationship between geographic features, but it is unlikely he/she needs the actual geographic space. It is better to err on the side of caution than to make a mistake that might lead to a breach in patient privacy and further restrict the access spatial researchers have to confidential data

## Methods

A map from the Front Page of the December 30, 2005 Baton Rouge *Advocate*, entitled "Deaths from Katrina hit both rich, poor", displayed a total of 412 mortality locations, though only 369 fell inside Orleans Parish which contains New Orleans. This map was scanned and georegistered using ArcMap 9.1. The process of georegistration, also called registering or rectifying an image, converts a representation of the earth into its real-world location by assigning coordinates to the image. After scanning the map, the image was added to an ESRI ArcMap 9.1 view already containing a shapefile of Census 2000 tract boundaries. On the *Advocate *map the point pattern of death locations is displayed on a choropleth map of poverty by census tract. Poverty is classified into four categories graded by colours from light yellow to dark brown (Figure 2). Due to the lack of streets or other geographic references that could be used in the georegistration of the map, only the Census tract layer was used as a source for control points assigned to the image.

### Georegistration

The 2000 Census tract boundary file provides intersections that are recognizable throughout the map and thus were the primary source for assigning geographic coordinates to the graphic. The accuracy of matching the image to its real-world location is dependent on assigning control points evenly throughout the map. In this case, due to reliance on recognizable tract boundaries, some areas were assigned more control points than others. Also, in the *Advocate *map, when contiguous Census tracts fall into the same poverty classification the boundary between them is no longer visible, thus degrading ability to use these areas for control points. Even with these potential sources of inaccuracy, the resulting overlay of paper map and digital tract boundary left little error.

### Digitizing death locations

Each mortality was heads-up digitized, meaning the mortality circle was added into the GIS by being drawn around its circumference using the mouse. Both this outer circle and the centroid, the circle's center point, were captured as digital layer files. The outer circle, once exported to Google Earth covered approximately one-and-a-half city blocks (Fig 2b). Each centroid was mapped onto a street map of New Orleans. Figure 2 shows the digitised centroids as red points on the original *Advocate* map.

### Employing digitized death locations in field analysis

From Arc 9.1, 8.5 × 11 size maps were generated for each neighborhood showing streets, street names and the digitised centroids. These maps were used by the field team who systematically went to each coordinate point on the map, estimating exactly where they should find the residence along the street section, including on which side of the road it should fall. The field team did not search for the mortality residence beyond the immediate vicinity of the dot on the map, unless the location was situated inside a city block with no indication as to which street section the residence fell. Those houses in which a mortality was marked by a search and rescue team were photographed, the address recorded, and a GPS coordinate captured, using a Trimble GeoExplorer 3 hand-held GPS receiver.

### Comparing field data with re-engineered death locations

The latitude and longitude coordinates of the re-engineered mortality and the verified address were displayed on high-resolution imagery (1 foot resolution post-Katrina imagery that originated with the Army Corps of Engineers and was flown by 3001 Inc.) of New Orlean**s **using ESRI ArcMap 9.1. In order to determine how close the re-engineered coordinates were to the Orleans Parish road network, the distance between each coordinate and the closest street section was recorded using the spatial join feature in ArcMap 9.1. The distance was also calculated between each pair of re-engineered coordinates and the field verified address. A second distance measure was also employed for these pairs being the number of separating houses between the re-engineered coordinate and the actual address. This count was easily achieved by using the high-resolution imagery.

In order to determine if the re-engineered mortalities had guided field teams to the verified residences or whether the discovery was by chance alone, one hundred simulation surfaces were created for each neighborhood. These simulation surfaces were comprised of randomly located residences, where the "n" for each neighborhood equalled the number of re-engineered mortalities extracted from the *Advocate *map (24 for New Orleans East, 20 for the London Canal area, and 36 for the Lower Ninth Ward). The simulation surface was created using Hawth's Analysis Tools for ArcGIS which provide additional functions to ESRI's ArcGIS program. The Generate Random Points tool was used to randomly distribute points across the polygon layer of Census tracts. In order to see how dissimilar a geocoded surface was to a randomly generated point surface in terms of mirroring the underlying street network, the distance between each randomly generated point and its closest street section was recorded using the spatial join tool in Arc 9.1. Similarly, to see how frequently a randomly generated point would fall closer to a field verified address than a mortality coordinate, the distance between the address and its closest randomly generated point was recorded using the spatial join tool in Arc 9.1. By recording this distance for 100 simulation runs, a test distribution of mortalities was created against which the distances of the mortality coordinate and field verified address pairs could be compared.

## Competing interests

The author(s) declare that they have no competing interests.

## Authors' contributions

AC JM and ML collected all field data. JM performed the re-engineering of mortality locations from the *Advocate *map. AC conceived of the study, and participated in its design and coordination and helped to draft the manuscript. All authors read and approved the final manuscript.

**Table 2:** Distance between re-engineered and field verified residences.

Distance in meters	0 to 5	6 to 10	11 to 15	16 to 20	21 to 25	Above 25
New Orleans East						
Percentage of matched	4.5	18.2	13.6	4.5	4.5	54.5
						
London Canal						
Percentage of matched	0	6.3	0	0	6.3	87.5
						
Ninth Ward						
Percentage of matched	0	0	19.2	3.8	7.7	69.2
